# The Frequency Spectral Properties of Electrode-Skin Contact Impedance on Human Head and Its Frequency-Dependent Effects on Frequency-Difference EIT in Stroke Detection from 10Hz to 1MHz

**DOI:** 10.1371/journal.pone.0170563

**Published:** 2017-01-20

**Authors:** Lin Yang, Meng Dai, Canhua Xu, Ge Zhang, Weichen Li, Feng Fu, Xuetao Shi, Xiuzhen Dong

**Affiliations:** Department of Biomedical Engineering, Fourth Military Medical University, Xi’an, China; University of Minnesota, UNITED STATES

## Abstract

Frequency-difference electrical impedance tomography (fdEIT) reconstructs frequency-dependent changes of a complex impedance distribution. It has a potential application in acute stroke detection because there are significant differences in impedance spectra between stroke lesions and normal brain tissues. However, fdEIT suffers from the influences of electrode-skin contact impedance since contact impedance varies greatly with frequency. When using fdEIT to detect stroke, it is critical to know the degree of measurement errors or image artifacts caused by contact impedance. To our knowledge, no study has systematically investigated the frequency spectral properties of electrode-skin contact impedance on human head and its frequency-dependent effects on fdEIT used in stroke detection within a wide frequency band (10 Hz-1 MHz). In this study, we first measured and analyzed the frequency spectral properties of electrode-skin contact impedance on 47 human subjects’ heads within 10 Hz-1 MHz. Then, we quantified the frequency-dependent effects of contact impedance on fdEIT in stroke detection in terms of the current distribution beneath the electrodes and the contact impedance imbalance between two measuring electrodes. The results showed that the contact impedance at high frequencies (>100 kHz) significantly changed the current distribution beneath the electrode, leading to nonnegligible errors in boundary voltages and artifacts in reconstructed images. The contact impedance imbalance at low frequencies (<1 kHz) also caused significant measurement errors. We conclude that the contact impedance has critical frequency-dependent influences on fdEIT and further studies on reducing such influences are necessary to improve the application of fdEIT in stroke detection.

## Introduction

Stroke is a severe cerebrovascular disease which can be clinically divided into two categories: ischemic and hemorrhagic stroke [1]. Stroke carries a high morbidity and mortality rate and has become the second leading cause of death worldwide [2]. Early and timely interventioncan significantly improve the prognosis of stroke patients. However, ischemic and hemorrhagic stroke patients require completely different treatments. Ischemic patients need to be treated with thrombolytic (clot-dissolving) drugs within 4.5-6h after the onset of stroke while the hemorrhagic stroke patients require emergency surgery [3]. So a prompt brain imaging for stroke detection should be performed before treatment. Unfortunately, in practice, it is impossible to obtain an urgent CT or MRI scanning in casualty wards or primary health care units[4]. Therefore, a portable and inexpensive medical imaging tool for rapid stroke detection is urgently needed. In such a case electrical impedance tomography (EIT) appears promising because the impedance of biological tissues differs for normal physiological and pathological states [5–7].

EIT is a functional imaging technology that is safe, portable, noninvasive and inexpensive. It aims to reconstruct the impedance (or change) distribution inside human body by injecting a set of currents through surface electrodes and measuring the boundary voltages resulting from internal tissues of the body [8]. According to the imaging principle, EIT can be categorized as static EIT, time-difference EIT (tdEIT) and frequency-difference EIT (fdEIT).

Static EIT reconstructs the absolute impedance distribution inside human body and is theoretically feasible in stroke detection. But it is limited by fundamental ill-posedness of EIT inverse problem and technical difficulties caused by unknown electrode-skin contact impedance, uncertainty in electrode positions and other systematic measurement errors [9]. Time-difference EIT, which produces time-difference images using boundary voltages measured at two different instants, has been successfully applied in long-term monitoring such as lung imaging [10]. But for stroke detection, time-referenced data before the onset of stroke are needed. In clinical practice, it is often difficult to acquire such data, making tdEIT inapplicable in stroke detection [11].

Lately, fdEIT, which recovers the frequency-dependent impedance changes by employing the differential results of boundary voltages simultaneously measured at different frequencies, was suggested to detect stroke because the impedances of stroke lesions and normal tissues exhibit distinct frequency-dependent properties [12, 13]. Although considerable advancements in fdEIT used for stroke detection have been made over recent years [5, 6], the effect of electrode-skin contact impedance on the boundary voltage measurement of fdEIT is still a challenging problem. Unlike tdEIT, which can theoretically eliminate the impacts of contact impedance under the assumption that conditions of measurement remain constant and the voltage changes are only attributed to the impedance changes of internal tissues over time[14], fdEIT is fundamentally unable to cancel out contact impedance by the simple subtraction of voltages because the contact impedance also changes with frequency. In actual practice of stroke detection using fdEIT, a wide frequency range (from 10 Hz to 1 MHz) is suggested to improve efficiency of fdEIT since the impedance of stroke lesion changes slowly with frequency [15]. Unfortunately, within this band, the contact impedance also varies greatly from several hundred kohms at 10 Hz to approximately 100 ohms at 1 MHz for a 1 cm^2^ area [16].

Therefore, it is imperative to investigate the effects of contact impedance on fdEIT used for stroke detection. Generally, the contact impedance adversely affects the accuracy of EIT measurement in two ways. On the one hand, contact impedance affects the current distribution beneath the electrodes, leading to the fact that the measurement results are determined not only by the internal tissue impedance but also by the contact impedance [17]. On the other hand, the imbalance of contact impedances between two measuring electrodes may cause the common-mode voltage to yield a differential-mode voltage at the amplifier input of the EIT system, which makes the measurement results contain the common-mode voltage [18]. In stroke detection using fdEIT, it is critical to know the degree of measurement errors or image artifacts caused by contact impedance.

Up to now, several studies have investigated the influences of contact impedance on fdEIT. Horesh *et al*. studied the influences of contact impedance on boundary voltages from 10 Hz to 2.5 MHz and pointed out the contact impedance could severely affect the accuracy of boundary voltages [7]. Malone*et al*. also investigated the effects of contact impedance on the reconstruction algorithm proposed by their group at 5 Hz-5 kHz [19]. In these studies, however, the contact impedances of all electrodes remained constant across frequency and the frequency spectral properties of contact impedance were not taken into account. Therefore, it is crucial to 1) investigate the frequency spectral properties of contact impedance on human head, and 2) quantify the frequency-dependent effects of contact impedance on boundary voltages and reconstructed images from 10 Hz to 1 MHz in stroke detection using fdEIT. To the best of our knowledge, no research has reported the systematic measurement of the frequency spectral properties of electrode-skin contact impedance on human head, as well as its frequency-dependent effects on fdEIT in stroke detection from 10 Hz to 1 MHz.

Accordingly, in this study, we first measured and analyzed the frequency spectral properties of electrode-skin contact impedance at different locations on 47 human subjects’ heads within 10 Hz-1 MHz. Second, according to the measurement results, we quantified the frequency-dependent effects of contact impedance on fdEIT used for stroke detection. Finally, in the discussion section, we suggested some approaches to reduce the impacts of contact impedance on fdEIT used for stroke detection in terms of data acquisition and reconstruction algorithm.

## Materials and Methods

### Frequency spectral properties of contact impedance on human head within 10 Hz-1 MHz

To investigate the frequency spectral properties of contact impedance on human head, we presented a new method to measure contact impedance of electrodes used in brain EIT (see S1 Appendix), which synthesized 4-electrode (4E) and 3-electrode (3E) techniques and was proven to be an accurate method for real head structure by numerical simulations and phantom experiments. This method was applied to measure the contact impedance on volunteers based on the 16 equidistant electrodes layout. Forty-seven normal adults served as subjects. The subjects were asked to hold still during measurement. The experiment was carried out after being approved by Ethics Committee of the Fourth Military MedicalUniversity. All volunteers signed the informed consentform.

**Fig 1:**
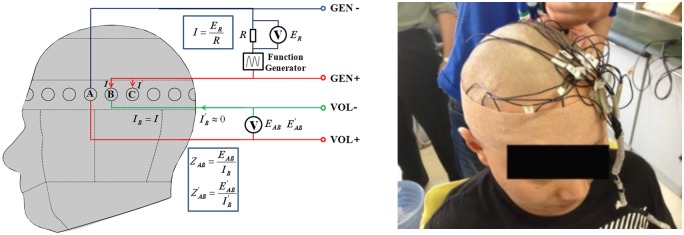
Measuring circuit of contact impedance. In this study, an impedance analyzer was used, and the voltage excitation mode (500 mV) was applied.

The measuring circuit is as shown in Fig 1. In this paper, 1260 Impedance/Gain-Phase Analyzer (Solartron Analytical, UK) with a 1294A interface was employed to conduct impedance measurement. For safety, the constant voltage exciting mode (500 mV) was applied across the electrodes while sweeping the frequencies from 10 Hz to 1 MHz in 51 steps. In all experiments of our study, weassured the subjects ahead that we would immediately terminate the experiment as soon as they felt uncomfortable.

All electrodes were Ag/AgCl powder (Oxford Instruments, Woking, UK) electrodes of 10 mm diameter. Before placing electrodes, the skin of all subjects was cleaned, and then the conductive gel (Elefix, Z-410CE, NIHON KOHDEN, Tokyo, Japan) was applied to ensure an optimal electrical contact between the electrode and the subject’s skin. For each subject, we measured the contact impedances of 16 electrodes that were equally spaced on the plane 2 cm above the supraorbital ridge. Electrode 1, 5, 9 and 13 were located above the left ear, between two eyebrows, above the right ear and the occipitalprotuberance, respectively. To keep the pressure between electrode and skin stable, a bandage (McDavid-4575, Bellwood, USA) with a length of 1.5 times the head circumference was wrapped around the head twice. Considering that many factors affect the measurement of contact impedance, such as temperature and sweat pores, the measurement was performed at 27±0.5°C and 15 minutes after installing all electrodes for measuring under stable condition.

The electrode-skin contact impedance consists of the impedance of three parts: the epidermis, the electrode-gel electrochemical reaction and the conductive gel [20]. To accurately model the electrode-skin contact impedance to assess its effects in later experiments, we measured the electrochemical impedance and conductive gel impedance by placing two electrodes face-to-face with the conductive gel in between and varying the thickness of the conductive gel (approximately 0.4 mm, which was twice as thick as a single electrode in practical application, and 1 mm). If ***Z***_**T**0.4_ and ***Z***_**T**1.0_ denoted the measured impedance when the thicknesses of conductive gel were 0.4mm and 1 mm respectively, the conductive gel impedance of single electrode could be obtained by (***Z***_**T**1.0_ − ***Z***_**T**0.4_) 0.2 / (1 − 0.4) and the electrochemical impedance by ***Z***_**T**0.4_ / 2 − (***Z***_**T**1.0_ − ***Z***_**T**0.4_) 0.2 / (1 − 0.4).

In this section, for each electrode, 47 sets of frequency properties of contact impedance were obtained.

### Frequency-dependent effects of contact impedance on the current distribution beneath the electrode

**Fig 2:**
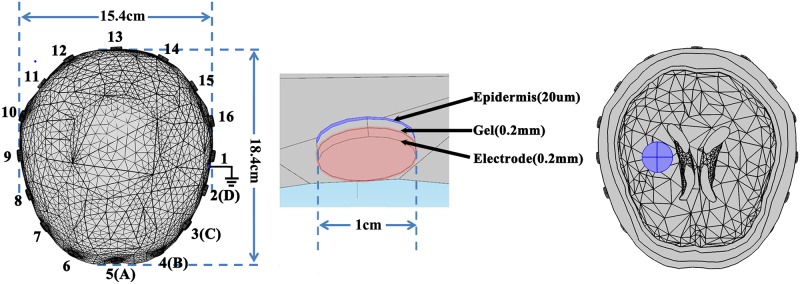
The realistic 3D head model with realistic electrode-skin interface including electrode, conductive gel and epidermis. A stroke lesion with 1.5 cm diameter was modeled.

**Fig 3:**
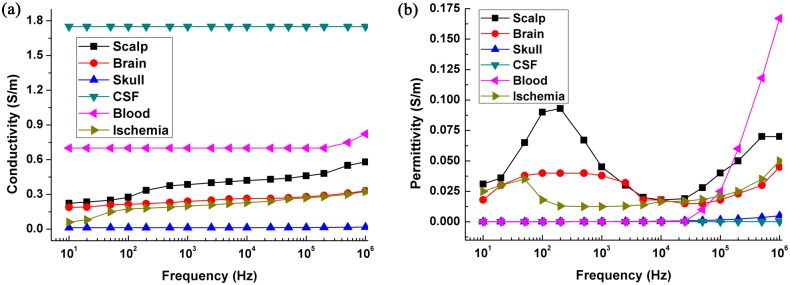
The dielectric parameters of head tissues, hemorrhagic tissue and ischemic tissue.

To evaluate the effects of contact impedance on the current distribution beneath the electrode, COMSOL Multiphysics 4.4 (Comsol Group, Sweden) was used to establish a realistic 3D head model based on the head CT images of one subject (Fig 2). In this model, the scalp, skull, CSF, parenchyma and ventricle were included, and the three components of electrode-skin contact impedance (epidermis, conductive gel and electrode) were also simulated by three cylinders. The conductivities and permittivity of these three components were calculated from the measured data, and the dielectric parameters of head tissues were based on the tissue data from Horesh *et al*.[12] and Tang *et al*.[21] (Fig 3). The number of elements in this 3D model was 280432, and a driving current of 100 μA was injected into the model. Sixteen electrode models were at the same positions as mentioned above.

As contact impedance greatly changed with frequency, the influences caused by contact impedance on the current distribution beneath the electrode were assessed from three aspects: the voltage in a single electrode, overall boundary voltages (16 electrodes, 192 measurements from opposite exciting-adjacent measuring mode) and reconstructed images.

#### Frequency-dependent effects of contact impedance on the boundary voltage in a single electrode

Six different magnitudes of contact impedance representing the whole range of contact impedance of Electrode 5 within 10 Hz-1 MHz (1.2×10^5^−8.2×10^5^*i* ohms, 1.7×10^4^−2.7×10^4^*i* ohms, 1.1×10^3^−3.1×10^3^*i* ohms, 4.2×10^2^−4.3×10^2^*i* ohms, 9.6×10–2.6×10*i* ohms, 6.5×10–1.2×10*i* ohms) were empirically selected to set the contact impedance of Electrode 5, whereas 500 ohms was selected for the rest of electrodes. Under the condition that Electrode 1 and 9 were the exciting electrodes and the impedances of head tissues within the model remained the values at 10 Hz,the current distribution beneath Electrode 5 was studied. Additionally, when the contact impedance of Electrode 5 gradually varied, the changes of absolute voltage in Electrode 5 were calculated.

#### Frequency-dependent effects of contact impedance on overall boundary voltages

In fdEIT, the boundary voltage difference between two different frequencies is of utmost importance. To evaluate the effects of contact impedance on stroke detection, the boundary voltage changes (*BVC*) caused by contact impedance was calculated and compared with *BVC* resulted from the stroke lesion within 10 Hz-1 MHz.

**Evaluation of the frequency-dependent effects of contact impedance on *BVC*:** To assess the effects of contact impedance on *BVC*, the 3D head model without the stroke lesion was used. First, the boundary voltages ${\bar{V}}_{n}^{f_{i}}$ were recorded in the case where the contact impedance of each electrode remained 500 ohms (equivalent to the impedance at 10 kHz, the median frequency of the whole frequency range), whereas impedance of the head tissues changed with frequency. From ${\bar{V}}_{n}^{f_{i}}$, we could obtain the boundary voltage changes ${\bar{V}}_{n}^{f_{i}}-{\bar{V}}_{n}^{f_{1}}$ caused by normal head tissues across frequency, where ***f***_1_ stands for the reference frequency (10 Hz, in this study).

Second, the impedance of normal head tissues changed with frequency, and three levels of contact impedance of each electrode (the minimum, mean and maximum of 47 sets of measured values of each electrode) were respectively chosen as the contact impedance of each electrode per frequency to calculate the boundary voltages $V_{n}^{f_{i},\delta }$, where *δ* represents the three levels of contact impedance: the minimum, mean and maximum. From $V_{n}^{f_{i},\delta }$, we could obtain the boundary voltage changes $V_{n}^{f_{i},\delta }-V_{n}^{f_{1},\delta }$ caused by normal head tissues and contact impedance. Finally, the *BVC* respectivelycaused by three levels of contact impedance were obtained using the Eq (1).

$$BVC^{f_{i},\delta }=\frac{\sum_{n=1}^{N}\left(V_{n}^{f_{i},\delta }-V_{n}^{f_{1},\delta }\right)-\sum_{n=1}^{N}\left({\bar{V}}_{n}^{f_{i}}-{\bar{V}}_{n}^{f_{1}}\right)}{\sum_{n=1}^{n}{\bar{V}}_{n}^{f_{1}}}\cdot 100\%$$ (1)

where ***N*** represents 192 measurements.

**Evaluation of the frequency-dependent effects of the stroke lesion on *BVC*:** To evaluate the effects of the stroke lesion (ischemic or hemorrhagic stroke) on *BVC*, the 3D head model with a lesion (a sphere with 1.5 cm diameter) to simulate ischemic or hemorrhagic stroke was employed. First, when the contact impedance of all electrodes was 500 ohms and the impedance of the stroke lesion remained the same as the value at 10 Hz (whereas the impedance of normal head tissues changed with frequency), the boundary voltages ${\bar{V}}_{n}^{f_{i}}$ were calculated. From ${\bar{V}}_{n}^{f_{i}}$, the voltage changes ${\bar{V}}_{n}^{f_{i}}-{\bar{V}}_{n}^{f_{1}}$ attributed to the normal head tissues over frequency could be obtained. Second, the boundary voltages $V_{n}^{f_{i}}$ were calculated when the impedance of stroke lesion also changed with frequency. From $V_{n}^{f_{i}}$, we could obtain the voltage changes across frequency $V_{n}^{f_{i}}-V_{n}^{f_{1}}$ caused by the stroke lesion and normal head tissues. Third, the *BVC* resulted from the stroke lesion were achieved by the eq (1).

#### Frequency-dependent effects of contact impedance on reconstructed images

In this section, the impedance changes caused by contact impedance and stroke lesion were reconstructed simultaneously using boundary voltage difference between two different frequencies. The damped least squares (DLS) algorithm was applied to reconstruct fdEIT images [22].

First, at all frequencies, the contact impedance of all electrodes was set as 500 ohms and the impedance of stroke lesions was initiated with the value at 10 Hz, while the impedances of normal head tissues varies with frequency increased. After that the boundary voltages ${\bar{V}}^{f_{i}}$ could be calculated accordingly. With respect to the reference frequency ***f***_1_, the boundary voltage changes ${\bar{V}}_{b}^{f_{i}}$ across frequency caused by normal head tissues could be computed by ${\bar{V}}_{b}^{f_{i}}=({\bar{V}}^{f_{i}}-{\bar{V}}^{f_{1}})$, where ***f***_1_ stands for 10 Hz.

Second, all of the electrodes except Electrode 5 were given a constant value of contact impedance (500 ohms) throughout the measurement frequency range, whereas the contact impedance of Electrode 5 was set to three levels (the minimum, the mean and the maximum of 47 sets of measured contact impedance at Electrode 5) per frequency, respectively. Additionally, the impedances of the stroke lesion and normal head tissues changed with frequency. Under the above conditions, boundary voltage $V^{f_{i},\delta }$ was calculated in three cases per frequency, where *δ* stands for one of three levels of contact impedance: the minimum, mean or maximum. Thus, the boundary voltage $V_{p}^{f_{i},\delta }$, which was jointly determined by normal head tissues, contact impedance of Electrode 5 and the stroke lesion, could be obtained by $V_{p}^{f_{i},\delta }=(V^{f_{i},\delta }-V^{f_{1},\delta })$.

**Fig 4:**
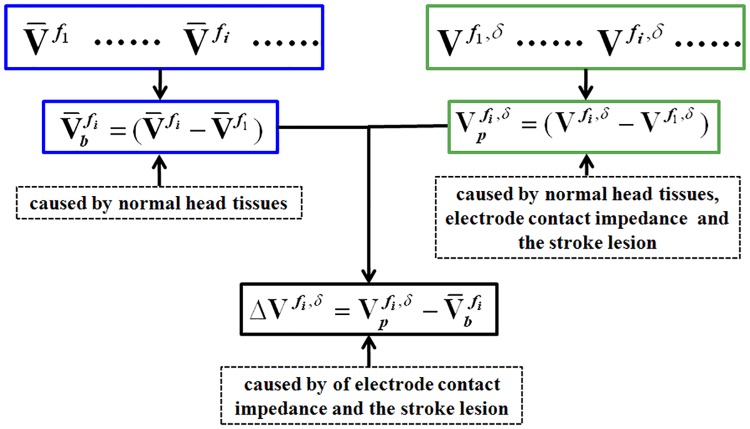
The procedure to obtain the boundary voltage difference for image reconstruction, where *δ* represents the different levels of effects of contact impedance and *f*_*i*_ denotes the frequency.

Finally, we reconstructed the image using $\Delta V^{f_{i},\delta }=V_{p}^{f_{i},\delta }-{\bar{V}}_{b}^{f_{i}}$, which represented the boundary voltage changes that arose from the stroke lesion and three different levels of contact impedance of Electrode 5. The flowchart that describes the calculation process of $\Delta V^{f_{i},\delta }$ is shown in Fig 4.

### Frequency-dependent effects of contact impedance imbalance on measurements

It is difficult to ensure the contact impedance balance between two measuring electrodes in practice, thus the existence of imbalance will lead to a conversion from common voltage to differential voltage at the amplifier input of the EIT system. The conversion can be denoted by [18]:

$$V_{D}=V_{CM}\frac{Z_{C-E1}-Z_{C-E2}}{Z_{I}}=V_{CM}\frac{\Delta Z_{E}}{Z_{I}}$$ (2)

where ***V***_***CM***_ is the common voltage on the two measuring electrodes, ***V***_***D***_ is the differential voltage converted from the common voltage, ***Z***_***I***_ is the average common-mode input impedance of the amplifier, ***Z***_***C*** − ***E***1_ and ***Z***_***C*** − ***E***2_ represent the contact impedance of two measuring electrodes respectively, Δ***Z***_***E***_ denotes the contact impedance imbalance between two measuring electrodes.

As we can see from Eq (2), the larger the unbalance Δ***Z***_***E***_ is, the greater the differential voltage ***V***_***D***_ converted from the common voltage ***V***_***CM***_ will be.

In this section, we evaluated the frequency-dependent effects of contact impedance imbalance between two measuring electrodes from two aspects: a single-channel measurement (two adjacent electrodes) and the reconstructed images. Because the input impedance (several Mohms) of the amplifier of our EIT system was quite large compared with the contact impedance (from several kohms to tens of ohms) at lager than 1 kHz, which would not produce severe effects on acquired data in theory [18], we only evaluated the effects of contact impedance imbalance at less than 1 kHz.

#### Frequency-dependent effects of contact impedance imbalance on a single-channel measurement

**Fig 5:**
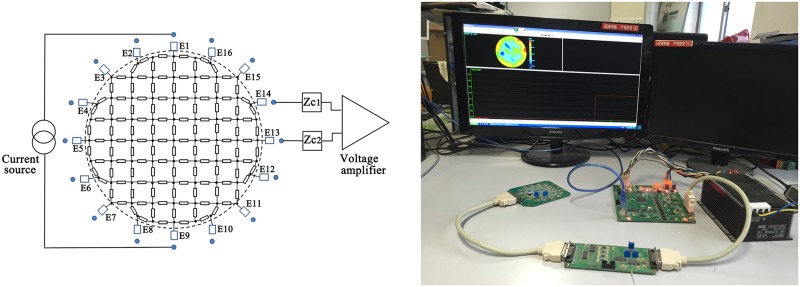
The circuit for assessing the effects of the contact impedance imbalance between two measuring electrodes, in which two resistors simulate the contact impedance.

We used the EIT system developed by our group to evaluate the influences of contact impedance imbalance on measurement within 1 kHz, which could operate at 10 Hz-300 kHz, with precision better than 0.05%. The current of 100 μA was employed. First, the input impedance of the amplifier and common mode rejection ratio (CMRR) were measured at several frequencies (10 Hz, 20 Hz, 50 Hz, 100 Hz, 200 Hz and 500Hz) within 1 kHz. Then, a resistor net model was connected with the EIT system (Fig 5). Third, because the contact impedance of Electrode 16 was greater than that of the other electrodes within 1 kHz, we selected frequency properties of contact impedance of Electrode 16 to evaluate the effects of contact impedance imbalance. A resistor with value equivalent to the maximum of the (acquired) 47 sets of contact impedance of Electrode 16 per frequency was in series with Electrode 16, whereas Electrode 1 was in series with resistors with values that were 100%, 90%, 75% and 60% of the maximum of 47 sets of contact impedance of Electrode 16 per frequency (60% of the maximum contact impedance of Electrode 16 approximated to the minimum contact impedance of Electrode 1). The boundary voltages $V_{\delta }^{f_{i}}$ between Electrode 16 and 1 were measured in these four scenarios per frequency. Finally, the measurement errors were calculated by comparing the voltages $V_{\delta }^{f_{i}}$ in three cases of 90%, 75% and 60% with the voltages $V_{100\%}^{f_{i}}$ in the case of 100%: $(V_{\delta }^{f_{i}}-V_{100\%}^{f_{i}})/V_{100\%}^{f_{i}}$, where *δ* represents 90%, 75% and 60%. These three cases of 90%, 75% and 60% represented three degrees of contact impedance imbalance between two measuring electrodes.

#### Frequency-dependent effects of contact impedance imbalance on reconstructed images

To assess the effects of contact impedance imbalance on image reconstruction, a hemispherical tank including a skull layer was utilized. First, according to the dielectric prosperities of scalp and brain parenchyma at six frequencies (10 Hz, 20 Hz, 50 Hz, 100 Hz, 200 Hz and 500 Hz), certain concentrations of saline was used to mimic scalp and brain parenchyma. Second, each of the 16 electrodes was connected in series with a resistor denoted by the number of the corresponding electrode, which meant Electrode 1 was connected in series with Resistor 1, etc. The resistance of all resistors except Resistor 1, 2, 16 was 500 ohms (independent of frequency), whereas the three remaining resistors had identical value that was frequency-dependent, which was determined by the maximum of the 47 sets of measured contact impedance of Electrode 16 per frequency. Third, agar cylinders were employed to simulate the ischemic stroke lesion. Only the case of ischemic stroke was studied here because for hemorrhagic stroke there will be no reconstructed frequency difference image below 1 kHz (in this frequency range the impedance of blood remains constant). The conductivity of the agar cylinder was in accordance with the dielectric properties of the ischemic tissue at 10 Hz. The agar cylinder was 6 cm in height and 1.5 cm in radius, and the immersion depth was 2.5 cm. The boundary voltages ${\bar{V}}^{f_{i}}$ were collected at the six selected frequencies. With respect to the reference frequency ***f***_1_, the difference of boundary voltages ${\bar{V}}_{b}^{f_{i}}=({\bar{V}}^{f_{i}}-{\bar{V}}^{f_{1}})$ could be calculated, which reflected the boundary voltage changes caused by normal tissues.

On the other hand, in order to simulate the frequency-dependent impedance of the ischemic tissue, agar cylinders with different conductivities were prepared according to the dielectric properties of the ischemic tissue at 20 Hz, 50 Hz, 100 Hz, 200 Hz and 500 Hz. When boundary voltages of a certain frequency were collected, the corresponding agar cylinder was used. Furthermore, to stimulate contact impedance imbalance, the resistance of Resistor 1 (in series with Electrode 1) changed three times at every frequency. It was respectively set to three levels, which were 90%, 75% and 60% of the maximum of the 47 sets of measured contact impedance of Electrode 16. At the six selected frequencies, the boundary voltages $V^{f_{i},\delta }$were collected, where *δ* stands for the three levels of contact impedance imbalance: 90%, 75%, and 60%. With respect to the reference frequency ***f***_1_, the difference of boundary voltages $V_{p}^{f_{i},\delta }=(V^{f_{i},\delta }-V^{f_{1},\delta })$ could be calculated, which reflected the boundary voltage changes caused by normal tissues, the ischemic tissue and contact impedance imbalance (three levels).

Finally, we acquired the image reconstruction using $\Delta V^{f_{i},\delta }=V_{p}^{f_{i},\delta }-{\bar{V}}_{b}^{f_{i}}$, which represented the boundary voltages changes resulted from the ischemic tissue and contact impedance imbalance (three levels). The procedure to obtain the boundary voltage difference $\Delta V^{f_{i},\delta }$ for image reconstruction is shown in Fig 4.

## Results

### Frequency spectral properties of contact impedance on human head within 10 Hz-1 MHz

**Fig 6:**
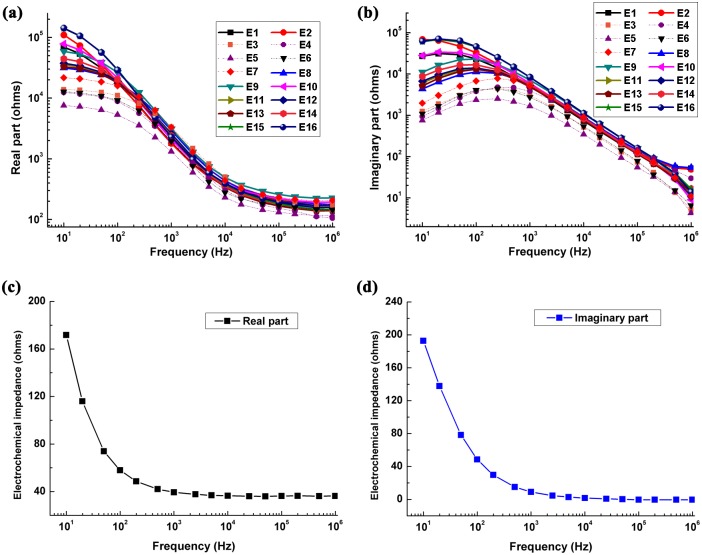
(a)(b) The contact impedance of all subjects at all electrodes. The dashed lines represent the measurement results of the electrodes at the forehead. (c)(d) The measurement results of electrochemical impedance and conductive gel impedance in the case of conductive gel with the thickness of 0.4 mm.

Fig 6(a) shows the mean value of contact impedance of all subjects at each electrode. The mean value of real part of measured impedance decreased rapidly below 10 kHz but slowly between 10 kHz and 1 MHz. The imaginary part was comparable to the real part below 10 kHz, suggesting that the imaginary part played an important role in measurement results below 10 kHz and should be separately considered in fdEIT. Furthermore, similar to the real part, the imaginary part varied greatly over frequency. However, the imaginary part had a small increase initially from 10 Hz to 200 Hz, before decreasing with frequency.

Additionally, the measurement results showed that the contact impedance of electrodes at the forehead is smaller than that at other sites, indicating that the contact impedance changed from site to site for a given subject. This might be because the epidermal layer at sites with no hair was thinner than that at other sites with hair. There were also differences in contact impedance at the same electrode for different subjects. For instance, in all measurement results of Electrode 16, the maximum and minimum of real part were 146 kohms and 30.6 kohms at 10 Hz, respectively.

Fig 6(c) shows the measurement results of electrochemical impedance and conductive gel impedance in the case of conductive gel with a thickness of 0.4 mm. The electrode-gel electrochemical impedance decreased with frequency (<10 kHz). At higher frequencies (>10 kHz), the real and imaginary parts tended to be constant at approximately 35 ohms and 1 ohm respectively, which demonstrated that resistance dominated in the electrode-gel interface.

### Frequency-dependent effects of contact impedance on the current distribution beneath the electrode

#### Frequency-dependent effects of contact impedance on the boundary voltage in a single electrode

**Fig 7:**
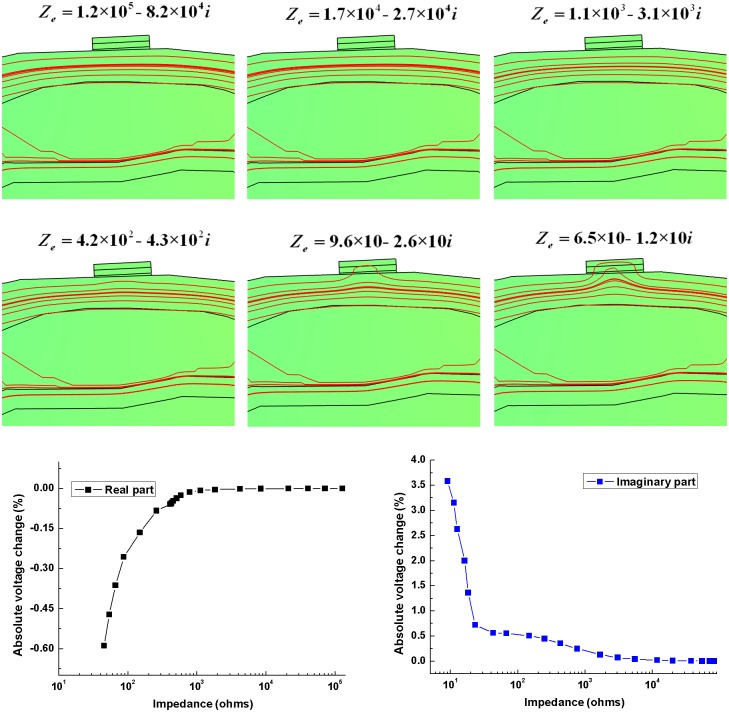
The current distribution beneath Electrode 5 with different contact impedances and the absolute voltage change in Electrode 5.

When the current was injected through Electrode 1 and 9, the current distribution beneath Electrode 5 in six cases where the contact impedance decreased gradually are shown as Fig 7. The current distribution was hardly impacted by the large contact impedance (>10 kohms in the real part) below 1 kHz and the influence on the current distribution increased with decreasing contact impedance. When the contact impedance decreased to 65 ohms in the real part, the current beneath Electrode 5 moved toward to the electrode. Therefore, a small contact impedance at high frequencies (>100 kHz) aroused a greater effect on the current distribution than a large contact impedance at low frequencies (<1 kHz). The absolute voltage change in Electrode 5 showed that the magnitude of the voltage change increased with contact impedance decreasing. The largest variation reached approximately 0.6% in the real part and 3.5% in the imaginary part.

#### Frequency-dependent effects of contact impedance on overall boundary voltages

**Fig 8:**
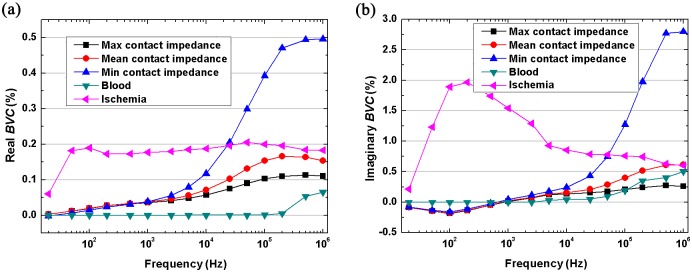
The boundary voltage changes (*BVC*) caused by three levels of contact impedance, ischemic stroke and hemorrhagic stroke.

Fig 8(a) shows the change to *BVC* caused by hemorrhagic stroke remained in a small range below 100 kHz. This may be because the conductivity of blood was almost unchanged (Fig 3). In contrast, there was an obvious variation in *BVC* above 500 kHz. However, the ischemic stroke led to a large change (about 0.2% in *BVC*) because the conductivity of ischemic tissue showed a large increase starting from 10 Hz (Fig 3). Furthermore, the *BVC* caused by contact impedance increased with frequency. The contact impedance gave rise to a small change (less than 0.05% in *BVC*) below 1 kHz, but the variation became larger than 0.1% above 100 kHz. In addition, the comparison of the effects from three levels of contact impedance reflected the fact that the smaller contact impedance had a larger influence on *BVC*. The *BVC* from the set of minimum contact impedance reached approximately 0.5% at 1 MHz, which was greater than the change caused by ischemic and hemorrhagic stroke.

In the imaginary part (Fig 8(b)), the results of *BVC* caused by hemorrhage, ischemia and contact impedance were similar to the real part. However, the magnitude of *BVC* in imaginary part was significantly larger than that in real part.

#### Frequency-dependent effects of contact impedance on reconstructed images

**Fig 9:**
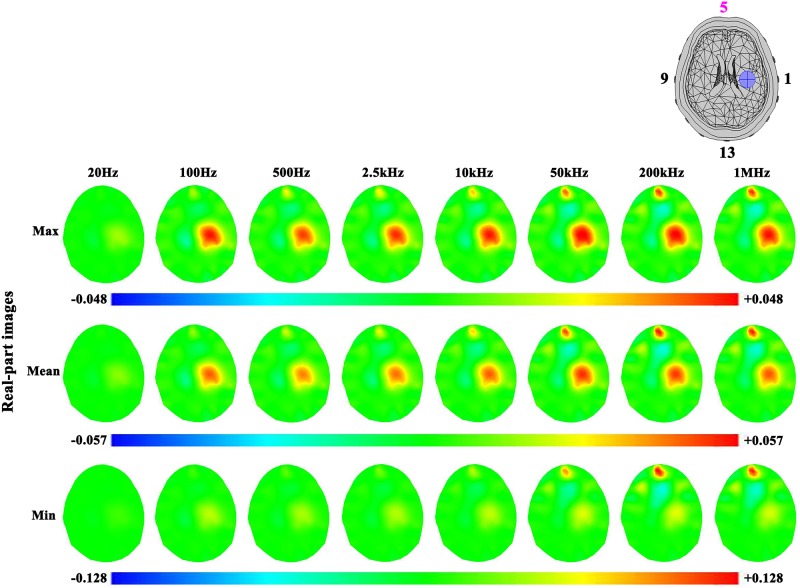
Real-part images of the ischemic stroke lesion and the effects of three levels of contact impedance (The reference frequency was 10 Hz). The physical unit of color bar is S/m in this study.

**Fig 10:**
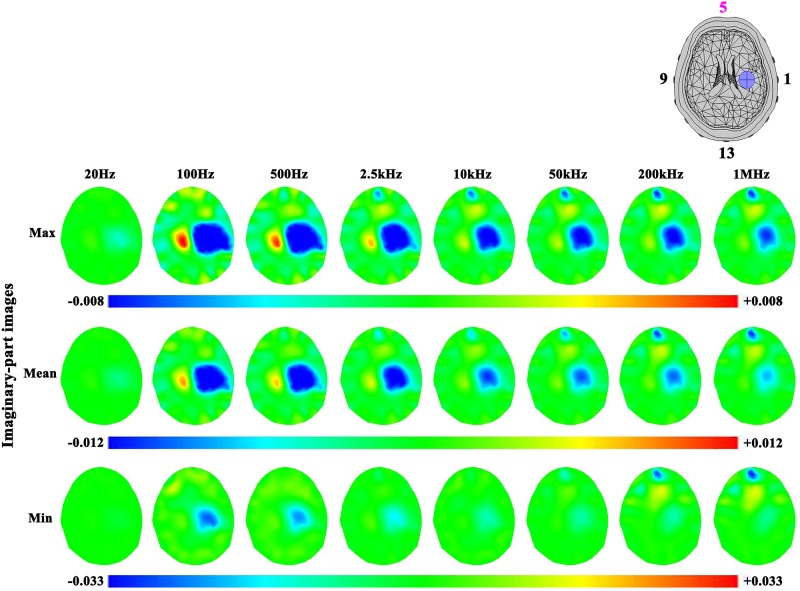
Imaginary-part images of the ischemic stroke lesion and the effects of three levels of contact impedance.

The reconstructed images were produced using the frequency spectral properties of the electrode-skin contact impedance on 47 human subjects’ heads. In the case of ischemic stroke (Fig 9), the real-part images showed that the artifacts under Electrode 5 caused by the contact impedance became more serious as frequency increased (the position of Electrode 5 is on the top of the reconstructedimages, as shown in Fig 9). For instance, the artifact in the image of the set of the minimum contact impedance made the stroke lesion less and less obvious with increasing frequency. Moreover, the smaller the contact impedance was, the more significant artifacts there were in reconstructed images. As an example, the reconstructed value of the artifact caused by the set of minimum contact impedance was greater than the maximum at the same frequency. In the imaginary-part images (Fig 10), the results were similar to the real-part images.

**Fig 11:**
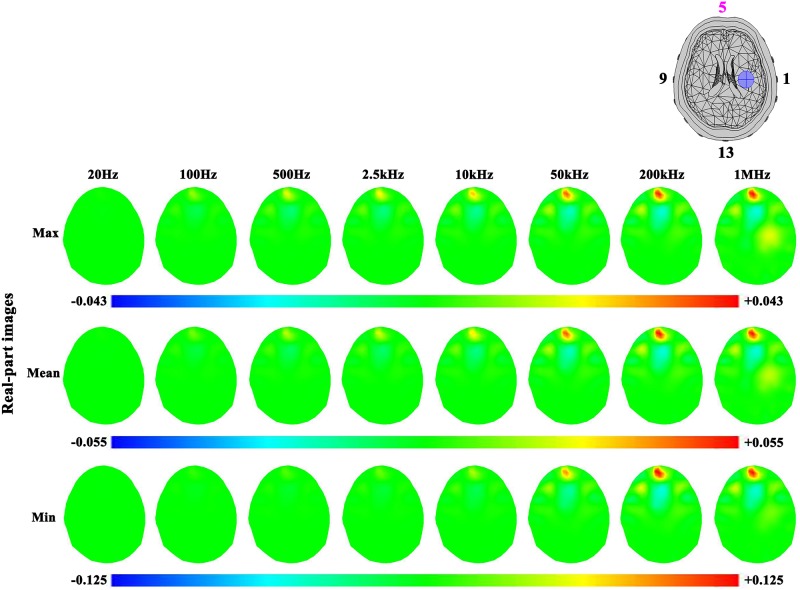
Real-part images of the hemorrhagic stroke lesion and the effects of three levels of contact impedance.

**Fig 12:**
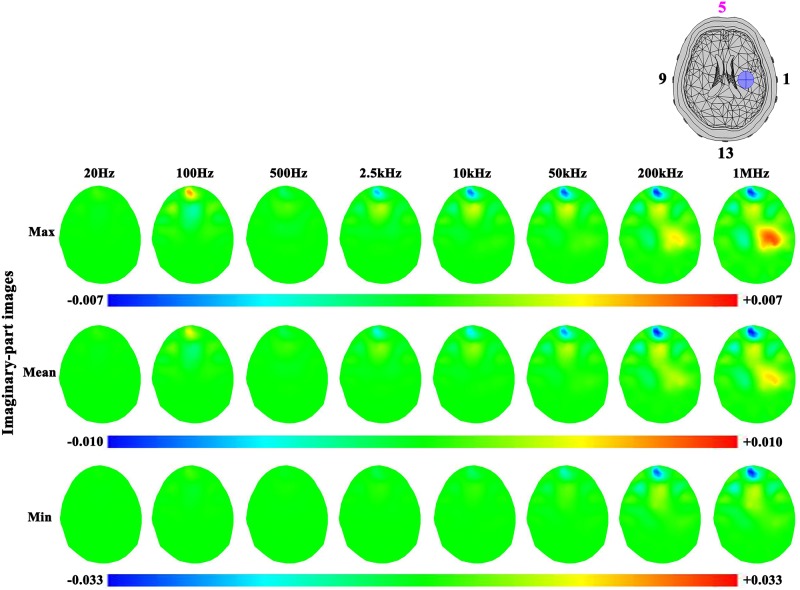
Imaginary-part images of the hemorrhagic stroke lesion and the effects of three levels of contact impedance.

In the case of hemorrhagic stroke (Figs 11 and 12), the results were similar to ischemic stroke. However, the hemorrhagic stroke was hard to be observed in the images (even in the real-part images at 200 kHz and 1 MHz), except the imaginary-part images of the maximum and mean sets of contact impedance at 200 kHz and 1 MHz. This is because the impedance of blood only changed at these two frequencies (Fig 3).

### Frequency-dependent effects of contact impedance imbalance on measurements

#### Frequency-dependent effects of contact impedance imbalance on a single-channel measurement

**Fig 13:**
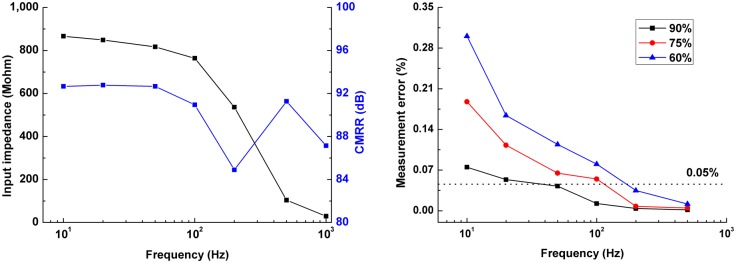
The effects of contact impedance on the boundary voltages because of imbalance between contact impedances.

Fig 13(a) shows that the input impedance and CMRR of our EIT system were larger than 20 Mohms and 70 dB within 1 kHz, which demonstrated that our EIT system had a strong ability to reject common-mode signalsin both input leads. Fig 13(b) showed that the greater the contact impedance imbalance between Electrode 16 and 1 was, the larger the measurement error was. For instance, the error was 0.075% at 10 Hz when the values of two resistors in series with Electrode 16 and 1 were 146 kohms and 131 ohms, while the error increased to 0.298% when the impedance of the resistor in series with Electrode 1 decreased to 87 kohms. In addition, it was also shown that the measurement errors decreased with frequency. The reason for this is that contact impedance imbalance (Δ***Z***_***E***_ in Eq (2)) between two measuring electrodes decreased significantly with frequency because the contact impedance (***Z***_***C*** − ***E***1_ and ***Z***_***C*** − ***E***2_) greatly reduced as frequency increased from 10 Hz to 1 kHz.

#### Frequency-dependent effects of contact impedance imbalance on reconstruction images

**Fig 14:**
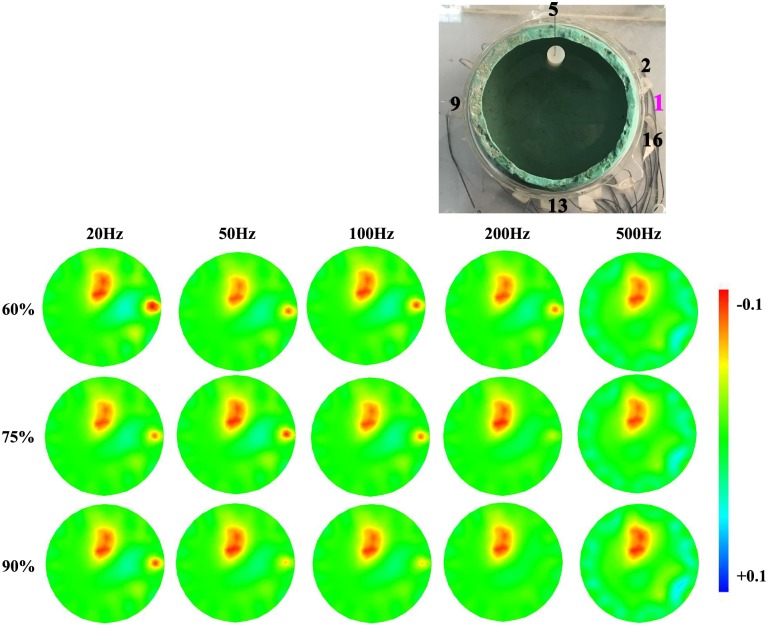
Reconstructed results of the ischemic stroke and the effects of the contact impedance. **The contact impedance imbalance was simulated by varying the resistor in series with Electrode 1**.

Fig 14 shows that the artifact caused by contact impedance decreased with frequency. Additionally, at the same frequency, such as 20 Hz, the artifact was more obvious in images corresponding to 60% of the maximum contact impedance than 90% of the maximum contact impedance, which indicated that the larger the imbalance (i.e. the smaller the percentage) of contact impedances between two electrodes was, the stronger the effects were. It should be noted that because the impedance of hemorrhagic tissue (blood) remained unchanged below 1 kHz, only the images that revealed the impacts of contact impedance on ischemic stroke lesion were displayed.

## Discussion

As previous studies have showed, fdEIT has potential application in stroke detection for its unique advantages of portability, fast imaging and no requirement of data at another time point. But fdEIT in stroke detection was inevitably affected by electrode-skin contact impedance because contact impedance varies significantly with frequency. Accordingly, in this study, for the first time we measured and analyzed the contact impedance of the brain EIT electrodes at different locations on 47 human subjects’ heads using the equidistant 16-electrode layout within 10 Hz-1 MHz. Then, according to the measurement results, we systematically evaluated the frequency-dependent effects of contact impedance in terms of the current distribution beneath the electrodes and the contact impedance imbalance.

### Frequency spectral properties of contact impedance on human head within 10 Hz-1 MHz

In our measurement results, the imaginary part played an important role in the contact impedance (Fig 6(a) and 6(b)), which may be caused by the epidermis because the imaginary part of impedance of conductive gel and electrochemical reaction is much smaller than that of contact impedance (Fig 6(c) and 6(d)). The epidermis is composed of many layers of compacted, flattened and non-nucleated dehydrated cells (called corneocytes) which are filled with cross-linked keratin, so the epidermis is relatively non-conductive [23]. However, the underlying layers of the epidermis are aqueous and conductive tissues. As a result, when the current is injected into human body through the surface electrodes (Ag/AgCl electrodes in our study), the epidermis presents high impedance against the transmission of the current, whereas it permits capacitive coupling between a metal electrode and its underlying conductive tissues[20]. Therefore, this capacitive coupling may account for a considerable portion in the imaginary part of contact impedance.

Low frequency currents (<1 kHz) are required to be used in fdEIT used for stroke detection, but such currents are more likely to cause skin sensation or pain, which makes the safety of applied currents an important issue. International Electrotechnical Commission (IEC) specifies a “patient auxiliary current” limit of 100μA from 0.1 Hz to 1 kHz [24], but IEC also points out that the application of larger currents is permitted for diagnostic purposes [25]. Considering the research conclusions of Romsauerove *et al*.[25], we used the constant voltage exciting mode (500 mV) to ensure safety in this study. Normally, with the smallest contact impedance the largest current would be obtained. Therefore, by selecting the smallest contact impedance at upper and lower limit of measurement frequency (6.6 kohms at 10 Hz and 1.8 kohms at 1 kHz, respectively)), we calculated the current to be 75 μA and 236 μA, which were still within the current limits (138 μA at 20 Hz, 207μA at 80 Hz and 280μA above 100 Hz) suggested by Romsauerova *et al*. for stroke detection using fdEIT. Most importantly, in all experiments of our study, weassured the subjects ahead that we would immediately terminate the experiment as soon as they felt uncomfortable. As expected, none of the subjects reported discomfort. Therefore, the constant voltage exciting mode (500 mV) we applied was safe.

### Frequency-dependent effects of contact impedance on fdEIT

In terms ofeffects of contact impedance on current distribution beneath the electrode, the results showed that the change in current distribution under the electrode increased with frequency (namely, decreasing contact impedance). This phenomenon might be interpreted by considering the contact impedance between the electrode and underlying tissue as the form of a conductor with certain impedance. In the case of large contact impedance at low frequency, the current distribution beneath the electrode was primarily determined by the impedance of the underlying tissues close to the electrode, and voltage at the electrode was approximately the average of the voltage of underlying tissues close to the electrode. In the case of small contact impedance at high frequency, both the underlying tissues and contact impedance determined the current distribution beneath the electrode, and the voltage at the electrode was thus strongly affected by contact impedance.

As for the effects brought by the contact impedance imbalance between two measuring electrodes, we found the measurement errors decreased with frequency. Therefore, a good measurement condition does not depend only on the contact impedances balance between two measuring electrodes but also on the individual electrode contact impedance. Accordingly, both improving the contact impedance balance and decreasing contact impedance are effective approaches to reduce the effects of contact impedance.

### Suggestions to reducing the effects of contact impedance on fdEIT in stroke detection

According to our findings, the electrode-skin contact impedance greatly decreased over frequency within 10 Hz-1 MHz. At low frequencies (<1 kHz), the contact impedance is very large (approximately 100 kohms) and may cause measurement errors due to the contact impedance imbalance between two measuring electrodes. Therefore, increasing the contact impedance balance is the most effective way to reduce measurement errors. However, in practice, it is very difficult to ensure this balance, which is determined by the skin preparation [20], electrode pressure and other variables. To improve the balance between contact impedances, a uniform electrode installation procedure, including cleaning the skin, applying conductive gel and using bandages, as we did in this study, is recommended. In addition, lowering the contact impedance to make it significantly smaller than the common-mode input impedance of the amplifier is also a useful approach to prevent converting common-mode voltages to differential-mode voltages, so some skin preparation techniques are suggested to perform before measurement, such as abrasion of the skin with some gels containing abrasives to remove a large proportion of the epidermis. Moreover, increasing the common-mode input impedance of the amplifier in the EIT system can also reduce the effects of the contact impedance imbalance on measurements [18].

At high frequencies (>100 kHz), the contact impedance is relatively small (approximately 100 ohms) and leads to measurement biases because the varying contact impedance with frequency affects the current distribution beneath the electrode. To mitigate the influences of contact impedance, attempting to simultaneously estimate the contact impedance and interior impedance of the object is a suggested approachbecause this approach seeks to separate the contact impedance and interior impedance. Using the complete electrode model (CEM), this goal can be achieved by selecting of regularization in image reconstruction. For the homogeneous medium in static EIT imaging, Vilhunen *et al*. [26] formulated the inverse problem as one of Bayesian estimation based on finite element approximation and Demidenko*et al*. [27, 28] relied on the analytical solution using a Neumann-to-Dirichlet matrix. Recently, focusing on tdEIT, Boverma*et al*. [29, 30] used linear-algebraic manipulations to simultaneously reconstruct time-varying images and the contact impedance, and the results showed that the artifacts due to contact impedance were successfully reduced using data from two humans. Similarly, for fdEIT, employing some form of regularization term in the inverse problem to simultaneously estimate the variation of contact impedance and interior impedance between different frequencies may alleviate the effects of contact impedance on images.

### Technical considerations in this study

In this study, we measured the contact impedance at different locations of human head based on the ring electrode configuration in which 16 electrodes were equally spaced in a circle. The validation of this electrode configuration in detecting the intracranial impedancehas been demonstrated by the large number of animal experiments [31, 32] and clinical studies[33, 34]. At present, for brain EIT, some studies have reported that the multiple-plane electrode configuration might enhance intracranial sensitivity and improve the image reconstruction performance in detecting intracranial lesions [35, 36]. But in theory, fdEIT with the multiple-plane electrode configuration still suffers from the effects of contact impedance in stroke detection. Additionally, according to our measurement results, the frequency spectral properties of contact impedance at different locations of human head are different (Fig 6). Therefore, if the multiple-plane electrode configuration is to be applied in stroke detection using fdEIT, it is a prerequisite to investigate the frequency spectral properties of contact impedance at different locations of multiple-plane electrode configuration and its effects on fdEIT in stroke detection.

Based on one subject’s CT images, we established a 3D head model to assess the effects of contact impedance on current distribution. In order not to lose generality, we selected the subject whose head parameters were closest to the average level, including head circumference (56.2 cm), length (18.4 cm) and width (15.4 cm). Nevertheless, as there exist inter-subject differences in head anatomical structure, the magnitude of effects of contact impedance on *BVC* and reconstructed images calculated based on this 3D model may be different with models based on other subjects’ CT images. However, we infer that the conclusions from the obtained results based on this model should agree with those based on other models.

## Conclusion

In order to better characterize contact impedance for improving the performance of fdEIT used for stroke detection, for the first time we measured and analyzed the frequency spectral properties of contact impedance on human head and systematically evaluated the frequency-dependent impacts of contact impedance on fdEIT in stroke detection from 10 Hz to 1 MHz. Our results showed that the large contact impedance at low frequencies (<1 kHz) could cause measurement errors due to the contact impedances imbalance and the small contact impedance at high frequencies (>100 kHz) could lead to measurement biases by changing the current distribution beneath the electrodes. Future studies will focus on alleviating the effects of the contact impedance from data acquisition and reconstruction algorithm. It is concluded that the contact impedance has severe frequency-dependent effects on fdEIT, and further studies on reducing its effects are necessary to improve the application of fdEIT in stroke detection.

## Supporting Information

S1 Appendix Measurement method of electrode-skin contact impedance. (DOCX)
