# Supplementary material for: The Frequency Spectral Properties of Electrode-Skin Contact Impedance on Human Head and Its Frequency-Dependent Effects on Frequency-Difference EIT in Stroke Detection from 10Hz to 1MHz
Source: PLoS One. 2017 Jan 20;12(1):e0170563. doi: 10.1371/journal.pone.0170563 (PMC5249181; doi:10.1371/journal.pone.0170563)
Supplement: S1 Appendix — (DOCX) [file pone.0170563.s001.docx]

**TITLE:** The frequency spectral properties of electrode-skin contact impedance on human head and its frequency-dependent effects on frequency-difference EIT in stroke detection from 10 Hz to 1 MHz

Supporting Information

**AUTHOR:** Lin Yang, Meng Dai, Canhua Xu, Ge Zhang, Weichen Li, Feng Fu, Xuetao Shi, Xiuzhen Dong

Department of Biomedical Engineering, Fourth Military Medical University, Xi’an, China

Email: [yanglin.0601@163.com](mailto:yanglin.0601@163.com)

**Supporting Information Appendix S1**

**Backgrounds**

In order to evaluate the frequency-dependent effects of contact impedance on fdEIT for stroke detection, an effective method to measure contact impedance of the brain EIT electrode is a prerequisite. At present, methods of measuring contact impedance can be mainly categorized into two classes: the methods based on the single-type electrode [1-4] and the approaches based on specially designed electrodes [5, 6]. On the one hand, although methods based on the single-type electrode (two-electrode technique [3, 4], three-electrode technique [1] and improved four-electrode method [2]) are conveniently implemented in practice, the measurement results do not include only the contact impedance but also unavoidably, the tissue impedance to a certain extent. It is thus presumed that the measurement results would be closer to the contact impedance if the tissue impedance could be more accurately estimated and subtracted in measurement results. On the other hand, the methods using specially designed electrodes precisely measure the contact impedance [5, 6]. However, for improving the accuracy of measurement, the design and instrumentation of electrode systems are quite sophisticated.

**Methods**

**Measurement method of electrode-skin contact impedance**

To accurately measure electrode-skin contact impedance, by investigating current paths within the head in a simulation, we proposed an accuracy-improved method based on the single-type electrode, which synthesized 4-electrode (4E) and 3-electrode (3E) techniques. Then, two comparison studies using a multi-layered tank were conducted to validate the proposed method.

**Principle of 4E-3E method**

The measuring circuit is shown in Fig 1(a). Assuming we were measuring the contact impedance of Electrode B, Electrode A and B were used to inject current$I$, and voltage$V_{\text{BC}}$between Electrode B and C was measured. The current through Electrode C was neglected due to the high input impedance of voltmeter, so$V_{\text{BC}}$was considered to be the sum of the voltage$V_{\text{Bcontact}}$caused by contact impedance$Z_{\text{Bcontact}}$of Electrode B and the voltage$V_{\text{BCtissue}}$attributed to the tissue impedance$Z_{\text{BCtissue}}$between Electrodes B and C, i.e. $V_{\text{BC}}=V_{\text{Bcontact}}+V_{\text{BCtissue}}$. Obviously, if we could obtain$V_{\text{BCtissue}}$,$Z_{\text{Bcontact}}$would be calculated from$V_{\text{Bcontact}}$by$Z_{\text{Bcontact}}=(V_{\text{BC}}-V_{\text{BCtissue}})/I$. However, since$V_{\text{BCtissue}}$cannot be measured directly in practice [7], a reasonable estimation method was required.

In this paper, before estimating$V_{\text{BCtissue}}$, the path of current flow within the head was investigated. A hemispherical 3D finite element model (FEM) with four layers (scalp, skull, CSF and brain) was established using COMSOL Multiphysics 4.4 (Comsol Group, Sweden), in which 16 electrodes were equally spaced on the scalp (Fig 1(b)). The outer radiuses of four layers of head tissues are 8.0 cm (brain), 8.2 cm (CSF), 8.7 cm (skull) and 9.2 cm (scalp) [8]. The impedances of the head tissues at 100 kHz were set into the model. The current distribution within the model is shown as Fig 1(c), in which the 1 mA current was injected through Electrode 1(A) and 2(B), which indicates that most of the currents flowed within the scalp underneath the electrodes close to exciting electrodes, such as Electrode 3 and 16. This was because the conductivity (0.0134 S/m) of skull was significantly lower than the scalp (0.46 S/m). Therefore, in Fig 1(a), the current$I'$flowing into the skull was trivial between Electrode B and C, which means that $V_{\text{BCtissue}}$was nonnegligible and should be reduced as much as possible from $V_{\text{BC}}$when measuring the contact impedance$Z_{\text{Bcontact}}$of Electrode B.

To select an optimal excitation mode that could guarantee that the current density between Electrode B and C was closest to the current density when Electrode A and B were the exciting electrodes, the voltages between Electrode B and C were compared under a series of different excitation modes. The results showed that $V'_{\text{BCtissue}}$ (which is the voltage caused by the tissue impedance between Electrode B and C) was most similar to$V_{\text{BCtissue}}$when the current was injected through Electrode A and D (Fig 1(f)). The current density is shown as Fig 1(d). As a result, in our method, $V'_{\text{BCtissue}}$was used to approximate$V_{\text{BCtissue}}$, i.e. $V_{\text{BCtissue}}\approx V'_{\text{BCtissue}}$. Furthermore, the contact impedance$Z_{\text{Bcontact}}$of Electrode B could be estimated by$Z_{\text{Bcontact}}=(V_{\text{BC}}-V_{\text{BCtissue}})/I\approx(V_{\text{BC}}-V'_{\text{BCtissue}})/I$.

**
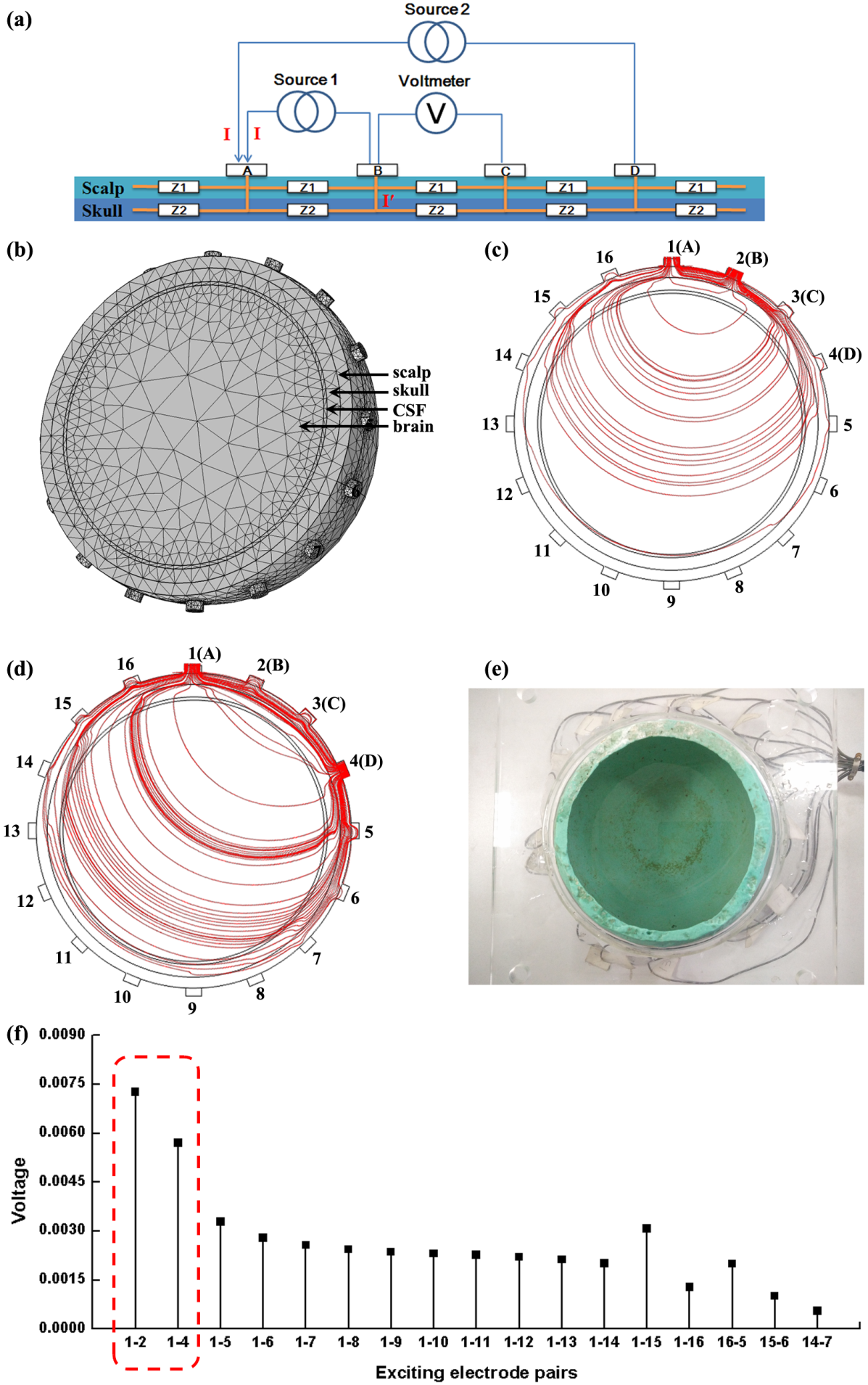
**

**Fig 1. (a) The measuring circuit of the 4E-3E method. $Z_{1}$and$Z_{2}$represent the impedance of scalp and skull between two electrodes. (b) A 3D hemispherical FEM model with scalp, skull, CSF and brain. (c) The current distribution when a 1 mA current was injected into the model through Electrode 1(A) and 2(B). (d) The current distribution when a 1 mA current was injected into the model through Electrode 1(A) and 4(D). (e) The 3D hemispherical tank with scalp, skull and brain. (f) The voltage between Electrode 2(B) and 3(C) under a series of different excitation modes.**

**Validation of the 4E-3E method in measuring contact impedance**

A hemispherical 3D tank with a skull layer (Fig 1(e)) was used to validate the method we present. The skull layer is made by plaster based on the real skull impedance at 100 kHz measured by Tang *et al.* and its conductivity is 0.01234 S/m [9]. Within the tank, the outer layer was filled with 0.257% saline and the inner layer with 0.146% saline to mimic the scalp and brain at 100 kHz. Sixteen resistors of 100 ohms (0.1% tolerance) were in series with each electrode to simulate the contact impedance. The contact impedance was measured by the two-electrode technique, the inner three-electrode technique (in which one measuring electrode is located between two exciting electrodes), the outer three-electrode technique (in which one measuring electrode is located outside of two exciting electrodes), the improved four-electrode method and the proposed method. The measurement results were compared with real contact impedance. We employed the EIT system developed by our group to acquire data at 100 kHz, which could operate at 10 Hz-300 kHz, with precision better than 0.05% and a common-mode rejection rate (CMRR) higher than 70 dB. Moreover, to further validate the 4E-3E method, three types of resistors (200 ohms, 500 ohms and 1 kohms) were in series with each electrode, and we compared the measurement results with different resistors.

**
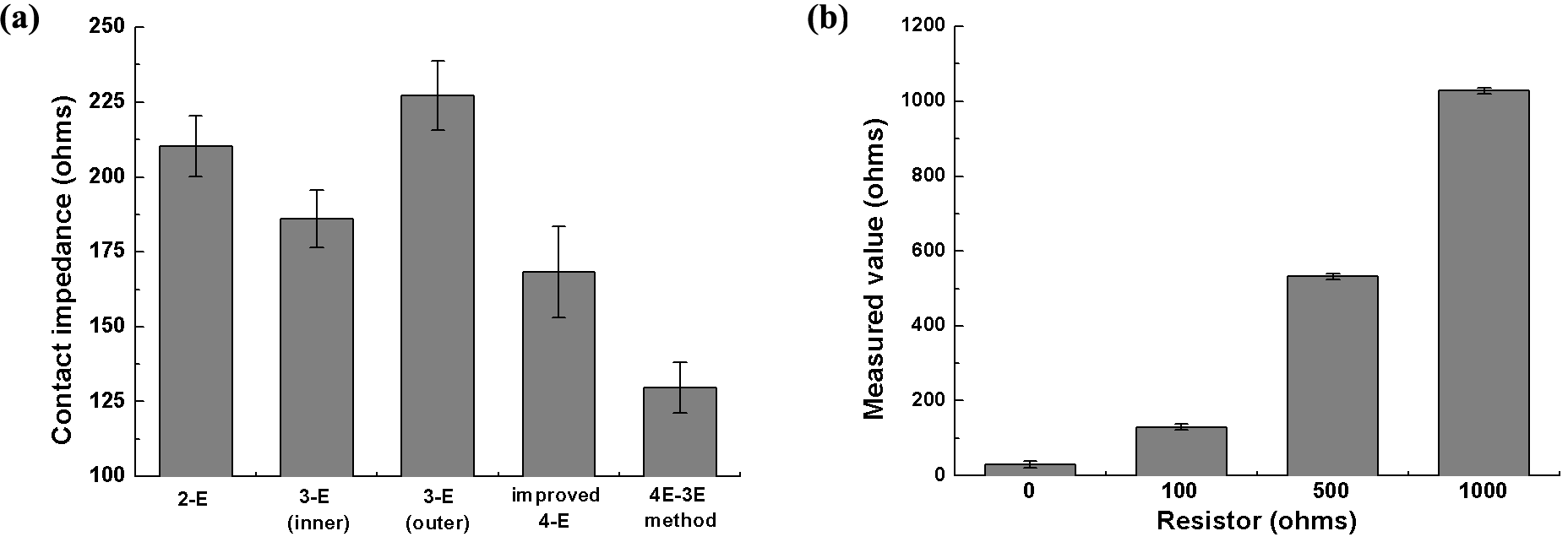
**

**Fig 2 (a) shows the contact impedance of 16 electrodes measured by different methods when the contact impedance was 100 ohms. The contact impedance (126.5±8.6 ohms) estimated by the proposed method was closest to the real one, suggesting that our method could reduce the tissue impedance in the measurement results to the greatest extent. Fig 2 (b) shows that when the contact impedance increased, the measured value by the proposed method could accurately reflect the increment. Thus, our method was a valid method to measure the contact impedance of brain single-type EIT electrodes.**

**Discussion**

In proposed 4E-3E method, we adopt an approximation method for measuring electrode-skin contact impedance. To obtain the best approximation that could guarantee that the current density between Electrode B and C was closest to the current density when Electrode A and B were the exciting electrodes, we compared the voltages between Electrode B and C under a series of different excitation modes. As we can see from Fig 1(c) and (d), the closer to the exciting electrode was, the greater the current density beneath the electrode was. Because the current density between Electrode B and C was large when the current was injected through Electrode A and B, a exciting electrode pair in which the exciting electrode were close to Electrode B and C should be investigated. Therefore, we mainly studied two cases. Case 1: we chose the Electrode 1 (closest to Electrode B and C) as one exciting electrode, and then Electrode 4, 5, 6 … and 16 were the other one respectively. The voltages between Electrode B and C under different exciting electrode pairs were measured and compared with$V_{\text{BCtissue}}$. Case 2: in theory, the voltage between Electrode B and C should be studied when Electrode 4 was the one excitation electrode and the rest of electrodes were the other electrode respectively. However, according to the reciprocity theorem, the results of the above two cases were similar. Therefore, in this study, we only showed the results of the first case. Additionally, we also studied some other exciting electrode pairs which are 16-5, 15-6, 14-7 and so on. But only the exciting electrode pairs were showed under which the voltages between Electrode B and C were greater.

The proposed 4E-3E method could reduce the tissue impedance contained in measurement results more than the other single-type electrode methods. Our method directly estimated the tissue impedance in the three-electrode technique by the measurement from the four-electrode technique. Though we chose an optimal excitation mode in which the current density in the four-electrode technique was closest to the current density in the three-electrode technique, the errors were still included in measurements due to the difference in current density between two modes. In practice, if the patients’ head CT or MRI images are available, we are able to obtain the relationship of voltages reflecting the current density in two excitation modes from simulation to further improve the accuracy of measurement.

In this study, the precision impedance analyzer was used to measure contact impedance rather than the EIT data acquisition system because our EIT system is able to operate with CMRR over 70 dB at less than 300 kHz but the precision significantly decreases beyond 300 kHz. However, by applying our method, the contact impedance of each electrode in the brain EIT electrode layout could be measured with the multi-frequency EIT system.

**References**

1. Spach MS, Barr RC, Havstad JW, Long EC. Skin-electrode impedance and its effect on recording cardiac potentials. Circulation. 1966;34(4):649-56. PubMed PMID: 5921760.

2. Xie L, Yang G, Xu L, Seoane F, Chen Q, Zheng L, editors. Characterization of dry biopotential electrodes. 2013 35th Annual International Conference of the IEEE Engineering in Medicine and Biology Society (EMBC); 2013 3-7 July 2013.

3. Mihajlovi V, x, Grundlehner B, editors. The effect of force and electrode material on electrode-to-skin impedance. 2012 IEEE Biomedical Circuits and Systems Conference (BioCAS); 2012 28-30 Nov. 2012.

4. Buxi D, Kim S, Helleputte Nv, Altini M, Wijsman J, Yazicioglu RF, et al. Correlation Between Electrode-Tissue Impedance and Motion Artifact in Biopotential Recordings. IEEE Sensors Journal. 2012;12(12):3373-83. doi: 10.1109/JSEN.2012.2221163.

5. Rosell J, Colominas J, Riu P, Pallas-Areny R, Webster JG. Skin impedance from 1 Hz to 1 MHz. IEEE Trans Biomed Eng. 1988;35(8):649-51. doi: 10.1109/10.4599. PubMed PMID: 3169817.

6. Woo EJ, Hua P, Webster JG, Tompkins WJ, Pallás-Areny R. Skin impedance measurements using simple and compound electrodes. Medical and Biological Engineering and Computing. 1992;30(1):97-102. doi: 10.1007/bf02446200.

7. Xu SW, Dai M, Xu CH, Chen CS, Tang MX, Shi XT, et al. Performance Evaluation of Five Types of Ag/AgCl Bio-Electrodes for Cerebral Electrical Impedance Tomography. Ann Biomed Eng. 2011;39(7):2059-67. doi: 10.1007/s10439-011-0302-9. PubMed PMID: WOS:000291479600018.

8. Ni AS, Dong XZ, Yang GS, Fu F, Tang C. Image reconstruction incorporated with the skull inhomogeneity for electrical impedance tomography. Comput Med Imag Grap. 2008;32(5):409-15. doi: 10.1016/j.compmedimag.2008.04.002. PubMed PMID: WOS:000257052500007.

9. Tang C, You FS, Cheng G, Gao DK, Fu F, Yang GS, et al. Correlation between structure and resistivity variations of the live human skull. Ieee T Bio-Med Eng. 2008;55(9):2286-92. doi: 10.1109/Tbme.2008.923919. PubMed PMID: WOS:000258722200019.
